# Blended learning models for introductory programming courses: A systematic review

**DOI:** 10.1371/journal.pone.0221765

**Published:** 2019-09-05

**Authors:** Ali Alammary

**Affiliations:** College of Computing and Informatics, Saudi Electronic University, Riyadh, Saudi Arabia; Swinburne University of Technology, AUSTRALIA

## Abstract

Teaching introductory programming courses is not an easy task. Instructors of introductory programming courses are facing many challenges related to the nature of programming, the students’ characteristics and the traditional teaching methods that they are using. Blended learning seems to be a promising approach to address these challenges. Many studies concluded that blended learning can be more effective than traditional teaching and can improve students’ learning experience. However, the current state of knowledge and practice in applying blended learning to introductory programming courses is limited. In an attempt to begin remedying this gap, this review synthesizes the different blended learning approaches that have been applied in introductory programming courses. It classifies them into five models then discusses the impact of each of these models on the learning experience of novice programmers. It concludes by providing some recommendations for instructors who want to blend their courses as well as some implications for future research.

## Introduction

Teaching programming to first year programming students is a difficult task for many instructors in higher education field. Novice programming students experience different types of difficulties which contribute to high dropout and failure rates in introductory programming courses [1, 2]. Traditional teaching approaches do not seem adequate in helping students to overcome these difficulties [3, 4]. Therefore, more instructors are realizing the need to develop and utilize new teaching and learning approaches that can better improve their students’ learning experience.

Over the last decade, blended learning has been growing in popularity as it has proved to be an effective approach to overcome various limitations related to traditional teaching approaches [5]. As result, a number of instructors have attempted to utilize blended learning to improve their students’ performance in introductory programming courses [6, 7]. In their attempts, they utilized different blended learning components and adopted different blended learning models.

However, it is still not clear if these blended learning models are appropriate for introductory programming courses and if one of these models is more appropriate than the other. This review provides a detailed examination of the different blended learning models that have been applied in introductory programming courses and outcomes associated with them. The review will draw on three theoretical foundations: constructivism [8], learning style theory [9], and technology integration in education [10]. The aim is to provide guidance for instructors of introductory programming courses by critically appraising and summarizing existing research. To the best of our knowledge, this is the first systematic review on this subject.

## Background

### Challenges of teaching introductory programming courses

Introductory programming is becoming a core course to many undergraduate degrees. However, teaching introductory programming courses is still challenging for most instructors [11, 12]. The literature shows many reasons for that. Some are related to the students’ characteristics, the teaching methods or the nature of programming [1, 13].

### Students’ characteristics

Different students have different learning styles and different preferences in the way they learn and acquire knowledge. The learning styles theory emphasizes that students learn more when the educational experience is geared toward their own learning styles [14]. Some students prefer to represent information verbally, while others prefer to do it visually. Some students prefer to learn in groups or with other people where others prefer to work alone and use self-study [15]. However, in traditional classrooms all students must learn in the same way and in accordance with the instructor’s style of teaching and pedagogical strategies.

Another challenge related to students’ characteristics is poor motivation. Many studies found that the majority of first year programming students do not have enough motivation to study programming, and that these students were most at risk of dropping out or failing introductory programming courses [16]. Traditional teaching approaches do not seem adequate to increase students’ motivation [1].

### Teaching methods

Traditional teaching methods are not very effective in supporting programming learning [2]. Instructors use most of class time to teach the syntax and semantics of a programming language. Less time is dedicated to monitor and enhance students’ problem-solving skills [16]. Traditional teaching methods are also not very helpful in engaging students in programming activities. According to Alturki [1], in order to make programming more appealing and interesting, instructors need to employ collaborative and student-centered teaching approaches.

### Nature of programming

Programming languages have complex concepts and syntax which are hard for most novice programmers to comprehend and apply in their own programming tasks [17]. In addition, learning to program requires a variety of skills such as critical thinking, abstraction and generalization [16, 18]. It also requires knowledge about programming languages, problem solving and programming tools [11, 19].

### Blended learning as an alternative to traditional teaching

Blended learning can be identified as the thoughtful integration of different online and face to face instructional methods such as: lectures, self-paced activities and online discussion groups [20]. A growing body of literature shows that blended learning can enhance students learning experience and overcome the shortcomings of traditional teaching approaches [4, 21, 22]. Blended learning can increase students’ flexibility and convenience, improve their learning outcomes and increase their engagement in learning [23]. It can also allow instructors to better interact with their students and develop variety of solutions to course problems [24].

However, it is important to note that only a thoughtfully planned blended learning approach can produce the richness and achieve the desired outcomes [20, 25]. Academics should try to maximize the benefit of traditional and online instructional methods by using each method for what it does best [26, 27].

### Components of blended learning

Alammary, Carbone [28] identified five different blended learning components. Two of them are face-to-face, while the other three are online components. These components were classified based on the type of interaction that each of them supports. Constructivist learning theory, the most influential theory impacting pedagogy and hence blended learning [29], suggests that knowledge does not exist independent of the learner; it is constructed through interaction with the content [30] or other individuals [31]. The five blended learning components are:

1. Face-to-face instructor-led: students attend a class where an instructor presents materials with little opportunity for interaction, hands-on learning or practice [32]. According to Griffin, Mitchell [33], this delivery method has two main pedagogical advantages: control (it allows instructors to maintain control over their students’ learning and tailor teaching strategies accordingly) and efficiency (it allows one instructor to deliver a large amount of content to a large number of students).
2. Face-to-face collaboration: educational approach that encourages students to work together in class, e.g., discussion groups, pair programming, problem-based instruction [34]. According to Sarason and Banbury [35] and Selvi and Perumal [36], face-to-face collaborative work can: (i) help students to construct a deeper understanding of the content being learned; (ii) better engage students; (iii) help students to develop critical thinking; and (iv) encourage students to take charge of their own learning.
3. Online instructor-led: instruction delivered online with an instructor who sets the pace and offers interaction, e.g., webcasts, virtual classrooms [32]. This component has the same benefits as face-to-face instructor-led with one extra advantage i.e., it is not constrained by location.
4. Online collaboration: educational approach that encourages students to work together online, e.g., online learning communities, online peer review [34]. Compared to face-to-face collaborative work, this method does not have the constraints of location and time.
5. Online self-paced: educational approach that allows students to study at their own pace, from their own location and in their own time e.g., online reading, watching videos [37]. Griffin, Mitchell [33] indicated four pedagogical benefits of online self-paced: (i) allowing students to choose the time most appropriate for their learning; (ii) allowing them to learn at their own desired speed; (iii) providing them with the flexibility to learn in any location; and (iv) allowing them to choose the most appropriate learning strategy.

### Utilizing systematic review

Several studies have applied different blended learning approaches to introductory programming courses. However, it is still not clear if a particular approach is more appropriate for introductory programming courses than the others. This systematic review will examine the different approaches to blended learning. The authors will then provide a classification of blended learning models and investigates the most appropriate model that can yield the maximum benefit for instructors. A systematic review was utilized to achieve this aim, as it is the most appropriate method when several empirical studies have been published in the area under investigation, but their results vary [38]. Systematic review is also useful in summarizing the current evidence within a specific domain and can improve accuracy of conclusions by showing whether findings across multiple studies are consistent and generalizable [39].

To identify relevant articles for inclusion in this review, several scientific databases were searched. The search terms used included: “programming” + “hybrid course”, “programming” + “blended course”, “programming” + “blended learning”, “programming” + “hybrid learning”, “programming” + “flipped classroom”, “programming” + “flipped class” and “programming” + “flipped course”.

In analyzing the extracted data, a thematic synthesis was conducted, taking an interpretive approach. Descriptive themes were drawn out of the extracted data followed by the development of several analytic themes [40].

## Methodology

This systematic review was conducted following the guidance provided by Liberati, Altman [41] and Kitchenham and Charters [42]. After finalizing the research questions of the review, a search protocol was identified. This protocol was necessary to minimize any possibility of research bias [42]. The protocol includes: (1) the inclusion and exclusion criteria for studies; (2) the scientific databases suitable for the review; (3) the search terms used to retrieve relevant studies; and (4) the methods for studies selection, screening, data extraction, and analysis. What follows is a detailed description of the applied steps.

### Defining the research questions

The first step of the systematic review process was to define the research questions. Based on the aim of this study, the following questions were defined:

1. What are the different blended learning models that have been applied in introductory programming courses?
2. How effective is blended learning in improving the learning experience of novice programmers?
3. Which model is the most appropriate for introductory programming courses?

### Criteria for including and excluding studies

This review examines studies of different designs. It includes qualitative, quantitative, and mixed methods studies. A study was chosen for the systematic review if it met the following inclusion criteria:

1. It has been conducted in the context of higher education. This means that the blended learning course presented in the study should have been taught to students in a higher education institution.
2. It discussed a blended learning approach for teaching an introductory programming course i.e. course that teaches the basics of programming to novice programmers.
3. The course presented in the article should have both face-to-face and online components. Some definitions of the term blended learning define blended learning as a mix of pedagogical methods both with and without the use of online components [43]. This paper adopts the most common and agreed upon definition of the term which stresses that a blended learning course should have a mix of face-to-face and online components [20, 44, 45].
4. The searches were not limited by publication date; however, the included studies were limited to the English language.

A study was excluded from analysis if it met one of the following exclusion criteria:

1. It was conducted in a non-higher education context e.g., K12 or corporate training.
2. Its focus is not discussing a blended learning approach for introductory programming courses.
3. The course presented in the article does not have a proper blend of online and face-to-face components i.e., totally online or totally face-to-face course.

### Search strategy

The third step of the process was to identify studies relevant to the review. Only electronic searches were performed using nine scientific databases: ACM digital library, ProQuest journals, Eric, IEEE Xplore, Sciencedirect, Taylor & Francis online, SAGE Journals, Computer database and Scopus. These databases were chosen because they are considered highly relevant for researchers in educational and information technology. The date of the last search was June 2019.

Population, Intervention, Comparison and Outcomes (PICO) proposed by Kitchenham and Charters [42] was used to construct the search strategy. At first, the major search terms were derived from the research questions.

- Population: introductory programming courses
- Intervention: blended learning

- Comparison and Outcomes: these two dimensions were not included in the search strategy as they were found to restrict the search too much and remove many relevant articles [46].

Then, synonyms for each search term were identified. After that, the Boolean OR was used to incorporate synonyms, while the Boolean AND was used to link the search terms from population and intervention. This resulted in the following preliminary search string:

("introductory programming" OR "first year programming" OR "novice programmers") AND ("blended learning" OR "hybrid learning" OR "blended course" OR "hybrid course")

After conducting an initial search, the search string was found to be too restrictive as it failed to find several relevant articles. Therefore, the population terms (i.e. "introductory programming", "first year programming", "novice programmers") were replaced by the term "programming". It was found that articles use too many terms to describe introductory programming courses and that only by scanning articles, researcher can decide if the investigated course is an introductory programming course or not. In addition, more synonyms for the intervention term (i.e. blended learning) were added to the search strategies. These synonyms were discovered while conducting the initial search.

The search strategies of all databases were checked and revised by an expert in electronic search strategies and according to his revision, the final search strategies were modified. Table 1 illustrates the final search strategies used to search the nine databases.

**Table 1:** Search strategies for databases.

Database	Search strategy
ACM digital library	((programming) AND ("blended learning" OR "blended course" OR "blended classroom" OR "blended class" OR "hybrid learning" OR "hybrid course" OR "hybrid classroom" OR "hybrid class"))
ProQuest journals	noft(programming) AND noft("blended learning" OR "blended course" OR "blended classroom" OR "blended class" OR "hybrid learning" OR "hybrid course" OR "hybrid classroom" OR "hybrid class")
Eric	programming AND ("blended learning" OR "blended course" OR "blended classroom" OR "blended class" OR "hybrid learning" OR "hybrid course" OR "hybrid classroom" OR "hybrid class")
IEEE Xplore	(("programming") AND ("blended learning" OR "blended course" OR "blended classroom" OR "blended class" OR "hybrid learning" OR "hybrid course" OR "hybrid classroom" OR "hybrid class"))
Sciencedirect	Title, abstract, keywords: programming ("blended learning" OR "blended course" OR "blended classroom" OR "blended class" OR "hybrid learning" OR "hybrid course" OR "hybrid classroom" OR "hybrid class")
Taylor & Francis online	[All: "programming"] AND [[All: "blended learning"] OR [All: "blended course"] OR [All: "blended classroom"] OR [All: "blended class"] OR [All: "hybrid learning"] OR [All: "hybrid course"] OR [All: "hybrid classroom"] OR [All: "hybrid class"]]
SAGE Journals	[All "programming"] AND [[All "blended learning"] OR [All "blended course"] OR [All "blended classroom"] OR [All "blended class"] OR [All "hybrid learning"] OR [All "hybrid course"] OR [All "hybrid classroom"] OR [All "hybrid class"]]
Computer database	“programming” AND ("blended learning" OR "blended course" OR "blended classroom" OR "blended class" OR "hybrid learning" OR "hybrid course" OR "hybrid classroom" OR "hybrid class")
Scopus	TITLE-ABS-KEY (“programming" AND ("blended learning" OR "blended course" OR "blended classroom" OR "blended class" OR "hybrid learning" OR "hybrid course" OR "hybrid classroom" OR "hybrid class" ) )

The search was limited to abstract, title, and keywords for ProQuest journals, ScienceDirect and Scopus. This setting was helpful in minimizing the number of irrelevant hits. The other databases were searched with the settings “all fields” since these databases do not allow a more precise configuration within their search queries.

### Study selection

After performing the search, all studies were entered into a Reference Manager System (EndNote) and duplicates were removed. Then, the titles and abstracts of the remaining studies were screened using the inclusion and exclusion criteria. Where a decision about inclusion could not be made, the full paper was read to make a definitive judgement. This process was revised by two independent reviewers, with results compared and discrepancies discussed until consensus was reached.

### Data extraction

In this step, data was extracted from the included studies using a data extraction form. The form was developed specifically for this review and was piloted on a sample of three papers. The form included seven items as shown in Table 2.

**Table 2:** Data extraction form items.

Data item	Description
Reference	Title, author, type (e.g. conference/workshop/journal), date
Aim	The study’s aim as stated by the authors
Approach	Blended learning approach applied in the study
Components	Blended learning components used in the course
Evaluation	Description of how the blended approach was evaluated
Outcome	Results of the evaluation
Comments	Remark about the study quality

### Quality assessment

Another important step of the review process was to appraise the methodological quality of the included studies. As the included studies vary in design, the quality assessment was performed by using quantitative, qualitative, and mixed-method critical appraisal tool developed by Rowe, Frantz [47]. The tool includes criteria for assessing the quality of all important aspects of research methodology [48, 49], including theoretical background, study design, data collection, data analysis, interpretation, and conclusions (see Table 3). For each criterion, a study scored either 1 (if criterion was met) or 0 (if criterion was not met). Total methodological quality scores were calculated by summing the individual criterion scores of each study. Methodological quality was considered “high” if the total score ≥ 4, “moderate” if the total score = 3, and “low” if the total score ≤ 2.

**Table 3:** Methodological quality appraisal tool.

Criterion		Score*
1. Outcome measures:		
A. Valid/reliable and well described?		1
B. Not valid/reliable, poorly described or not identified?		0
2. Background/literature review:		
A. Detailed?		1
B. Limited?		0
3. Sample:		
A. Well described?		1
B. Poorly described?		0
4. Study design or methodology:		
A. Clear?		1
B. Not clear?		0
5. Conclusions:		
Supported by the study results?		1
Not supported by the study results?		0
**Methodological quality:**		
**High**	**Moderate**	**Low**
Total score ≥ 4	Total score = 3	Total score ≤ 2

* Total score = sum of individual scores

Again, this step was performed by the author and two independent reviewers. They first worked independently and then discussed the results until they reached agreement. Given the lack of consensus about the role of quality assessment as part of systematic reviews, no article was excluded on the basis of its methodological quality. The quality appraisal served to gain an understanding of the relative strengths and weaknesses of the included studies. In addition, as noted by many scholars, studies of poorer quality tend to contribute less to the synthesis [50–53].

### Synthesis of results

After quality appraisal, data analysis of the studies was performed. The extracted data were analyzed using a narrative format according to pre-determined themes emerged from the research questions. The themes included: blended learning approach, blended learning components, evaluation methods, outcomes of applying the evaluation.

## Results

**Fig 1:**
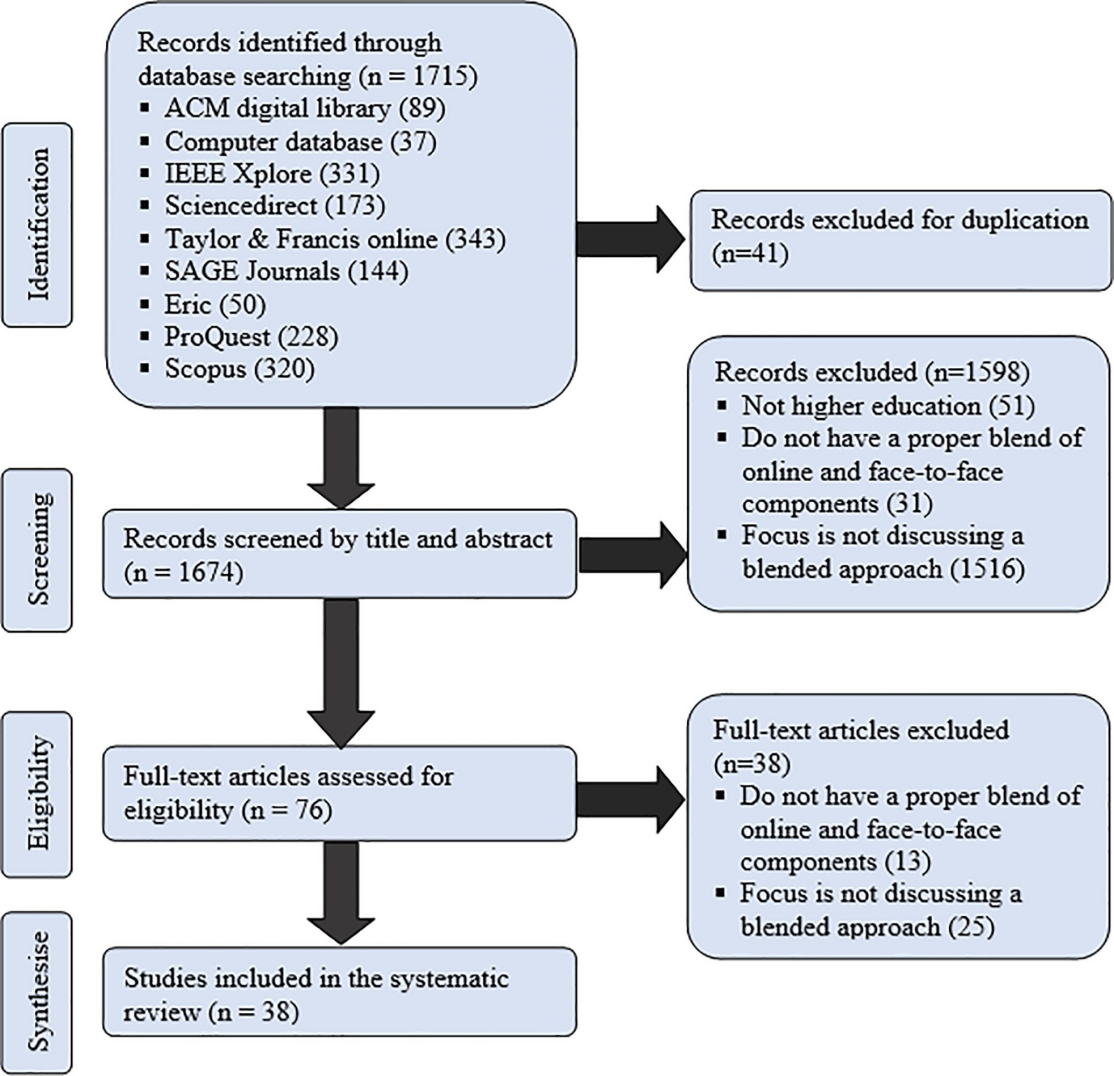
Flowchart of the systematic review process.

A total of 1715 records were retrieved from the nine electronic databases. Forty-one of them were excluded for duplication. Then, 1598 were eliminated when reading titles and abstracts. The full text of the remaining 76 articles was retrieved for full review. Thirty-eight of them were deemed relevant and were included in this systematic review. For the full results of the review process, see Fig 1.

### Publication year and geographic distribution

**Fig 2:**
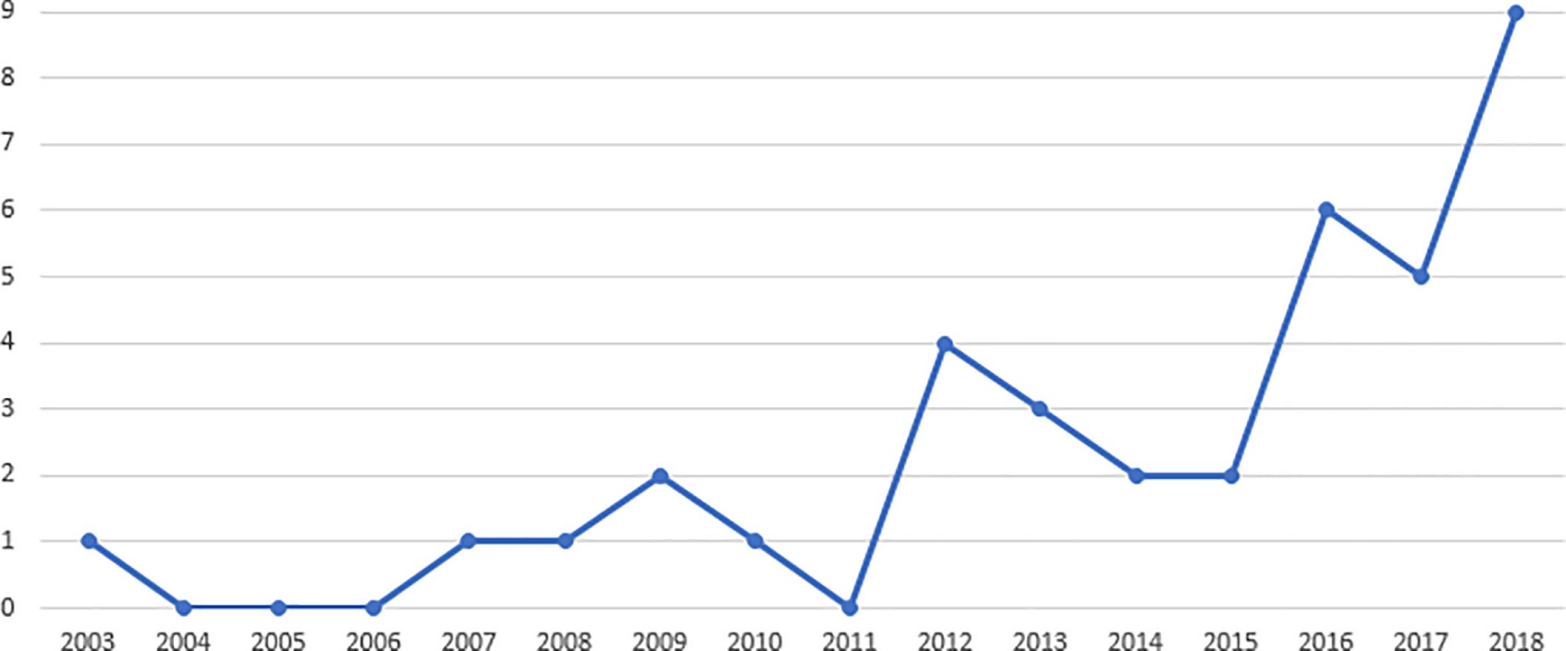
Publication year of the included studies.

The distribution of studies by publication year is presented in Fig 2. As can be seen, the largest number of these studies were published in the last three years (20 studies, 53%). However, even though the term blended learning has been in use since 1999, only five papers (13%) were published before 2010.

It is important to note that one study published in the first half of 2019, when data collection ended, are included in this review but was not reported in Fig 2. This is because it does not represent an accurate picture of the whole year of 2019.

**Fig 3:**
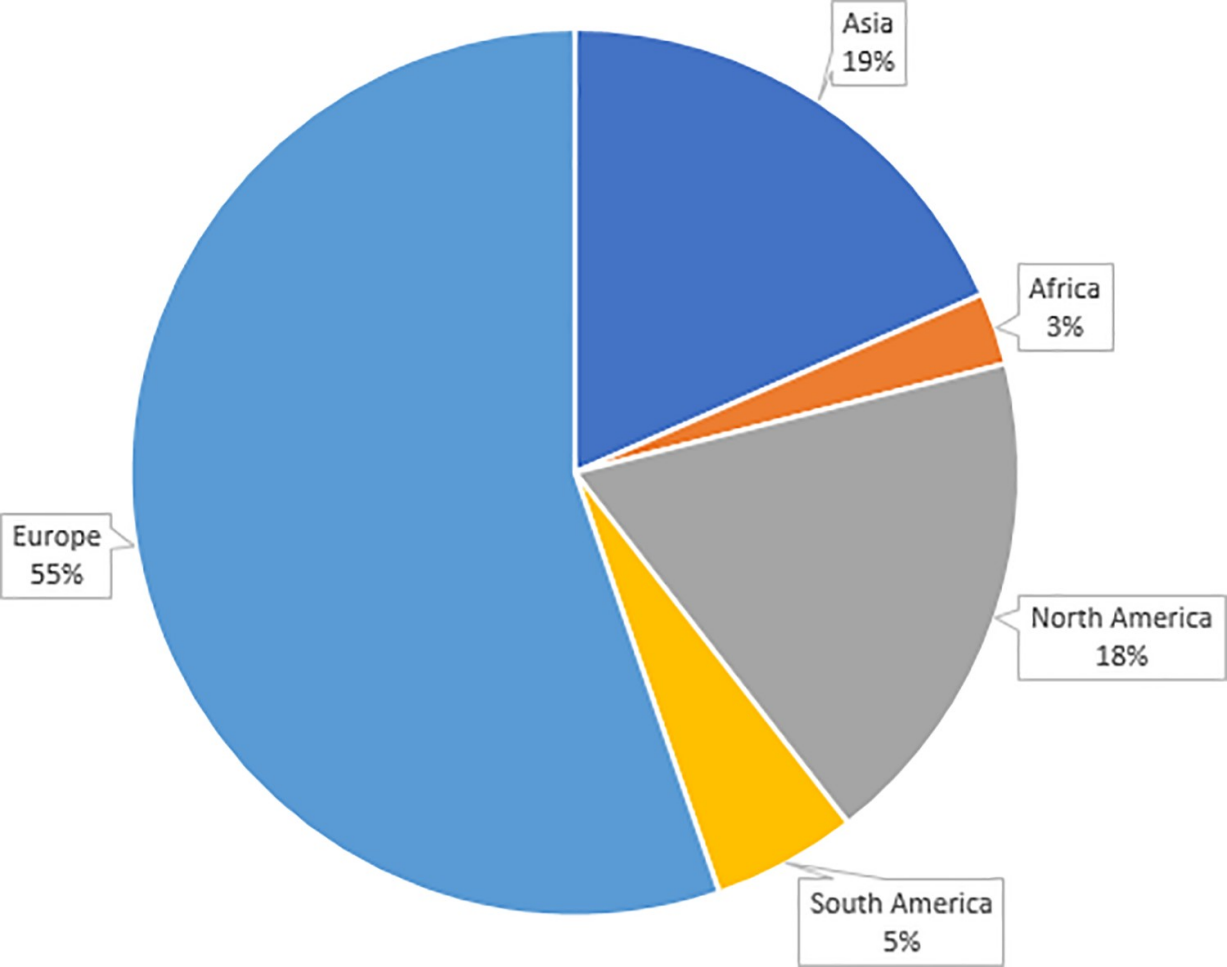
Geographic distribution of the included studies.

Fig 3 shows the geographical distribution of the included studies. The majority of these studies (21 studies, 55%) were published in Europe (Turkey = 8, Germany = 2, UK = 2, Spain = 2, Serbia = 2, Switzerland = 1, Norway = 1, Romania = 1, Croatia = 1, Sweden = 1). After Europe, the most common publication areas were North America with 7 studies (USA = 5, Canada = 1, Mexico = 1) and Asia with 7 studies also (Kazakhstan = 2, Thailand = 1, Taiwan = 1, Qatar = 1, Hong Kong = 1, China = 1). The remaining three studies were from South America (2 studies) and Africa (1 study).

### Quality assessment

As has been explained in the methodology section, a quality appraisal tool comprising five criteria was used to assess the quality of the included studies. A summary of the methodological quality appraisal is presented in Table 4. The overall methodological quality of the studies was good. More than half of them (52%) met all the five quality appraisal criteria. Another eight studies (21%) met four of the five criteria. Only three studies (8%) had major methodological quality issues i.e., met only one quality criterion.

**Table 4:** A summary of quality appraisal, by criterion.

Criterion	Quality assessment of studies	
	Met criterion	Did not meet
Outcome measures	28	10
Background/literature review	37	1
Sample	22	16
Study design or methodology	29	9
Conclusions	35	3

The most unmet criterion is the one related to study sample. Sixteen studies (42%) were found to poorly describe their sampling strategies. Ten studies (26%) had issues with their outcome measures. However, as discussed before, no study was excluded on the basis of quality. The detailed quality appraisal of all studies is included as supplemental material (S1 Table).

### Blended learning approaches

Table 5 summarises the different blended learning approaches that have been applied in the reviewed studies. A total of forty approaches were identified in these studies, as two studies [54, 55] presented two different approaches each. Ten studies (26%) applied an already existing approach (i.e., flipped classroom), while the rest (74%) developed their own approaches.

**Table 5:** Approaches applied by the reviewed studies.

Study	Approach
Albrecht, Gumz [56]	• Course content is delivered online • An online tool for assessing programming exercises is used for students to practice coding • Optional labs are used to provide feedback and support to the students
Alhazbi [57]	• Course content is delivered through lectures and self-based online resources • Online weekly formative assessments are used to provide students with feedback regarding their understanding of the subject • In labs, students work on coding exercises • Face-to-face and online collaborative activates are added for students to discuss several programming problems
Alonso, Manrique [58]	• Content delivery and practical coding activates take place online (via videoconferencing and self-paced activities) • Two face-to-face classes are used to introduce students to the course and evaluate their progress
Álvarez, Martín [59]	• Course content is delivered through lectures • An online learning environment is also used for content delivery and programming practice
Bati, Gelderblom [6]	• Lecture time is spent on content delivery, live coding and problem solving • Online resources are added for students to study on their own pace • Labs involve pair programming using questions from the online resources
Băutu, Atodiresei [60]	• Course content is delivered through lectures while labs focus on coding and problem solving • Online content is added as supplementary materials for students to study on their own pace • Students are also required to use online tools to share and discuss their own ideas with their colleagues
Bi and Shi [61]	• Course content is delivered online • Class time is spent on explaining difficult concepts and answering students’ questions • Students use the Moodle platform to discuss various programming topics with their peers
Boyle, Bradley [62]	• Course content is delivered through lectures and self-based online resources • In labs, students work on programming exercises
Breimer, Fryling [54]–Half Flip	• Course content is delivered through lectures and self-based online resources • Half of the lectures are used to complete coding exercises extracted from the online resources, while the other half are used for traditional lecturing
Breimer, Fryling [54]–Full Flip	• Half of the lectures are replaced by online self-paced activities • The remaining lectures include practical coding activities directly related to the concepts covered on the online materials
Cabrera, Villalon [63]	• Course content is delivered through lectures and self-based online resources • Variety of online and face-to-face collaborative activities are added for students to practice coding
Cakiroglu [64]	• The traditional face-to-face lectures are replaced by virtual classes • In labs, students work on programming exercises • Online self-paced are added as supplementary materials
Chen, Li [65]	• Course content is delivered through lectures • Out-of-class, students work in small groups on coding exercises • Online learning environment is used for students to discuss with peers and share knowledge
Clark, Besterfield-Sacre [66]	• The traditional face-to-face lectures are replaced by online self-paced learning • Labs hours are spent on collaborative activities that focus on coding and problem solving
Davenport [67]	• Course content is delivered online (reading, short videos) • In-class time is spent on coding and problem solving both individually and collaboratively as needed
Dawson, Allen [4]	• Course content is delivered mainly online with weekly mini-lecture to address questions raised by students in their pre-class work • Students spend the majority of lecture time on collaborative activities that focus on coding and problem solving
Deperlioglu and Kose [68]	• Course content is delivered face-to-face and through online learning resources • Students practice coding both face-to-face and online • Class time is spent on explaining difficult concepts and answering students’ questions • Students are also required to use an LMS to discuss various programming topics with their peers
Hadjerrouit [69]	• Course content is delivered through lectures and self-based online resources • In labs, students work individually or in small groups on coding exercises • Online collaborative activates are added for students to discuss their programming solutions
Hauswirth and Adamoli [55]—Asynchronous	• Course content is delivered online • In-class time is spent on checking students’ mastery of the weekly topics and providing them with feedback
Hauswirth and Adamoli [55]–Synchronous	• Course content is delivered through lectures while labs focus on coding and problem solving • Online content is added as supplementary materials for students to study on their own pace
Impelluso [70]	• Course content is delivered in class (algorithm and syntax were taught via Face-to-face lectures) and via online resources (a series of MPG videos) • Coding and problem solving took place online (Wimba software) as well as face-to-face in order to seek help from instructor
Jonsson [71]	• Course content is delivered online (short videos) • Lab time is spent on coding and problem solving • Problem sessions are setup up for students to work on programming problems in small groups
Ortíz-Ortíz, Jiménez-Murillo [72]	• Course content is delivered through lectures while labs focus on coding and problem solving • An online tool was used to support students learning by allowing them to learn programming concepts at their own pace, anytime from anywhere
Othman, Pislaru [73]	• Course content is delivered through face-to-face lectures, online virtual classes and self-based online resources • Face-to-face tutorials are conducted to allow students to practice coding • Group discussion is facilitated by using variety of online tools (wiki, blogs and discussion board)
Özyurt and Özyurt [74]	• Course content is delivered online (Facebook group) • In-class time is spent on coding and problem solving (in computer lab)
Djenic, Krneta [75]	• Course content is delivered through lectures and self-based online resources (online multimedia textbook) • Students practice coding in the lab • Additional practical exercises and knowledge assessments were regularly given over the Internet • Online discussions were added for students to communicate with each other
Šarić and Šerić [76]	• Students use an online system (AC-ware Tutor) to learn conceptual knowledge • Face-to-face lectures are spent on clarifying and applying the conceptual knowledge. • Lab time is spent on coding and problem solving
Djenic and Mitic [77]	• Course content is delivered through lectures • face-to-face collaborative activities are added for students to practice coding and problem solving • Online self-paced is also used for students to practice coding by using variety of tools
Sun, Kindy [78]	• Course content is delivered online (audio over PowerPoint slides, self-assessment quizzes, and Facebook group) • Lab time is spent on coding and problem solving (individually or with collaboration with other students)
Timmermann, Kautz [79]	• Online self-paced activities replace some face-to-face classes • Course content is delivered through lectures and self-based online resources • Lectures are changed to focus on algorithms and data structures • An online tool for assessing programming exercises is used for students to practice coding
Tritrakan, Kidrakarn [80]	• Course content is delivered mainly through self-based online resources • Difficult concepts are explained in the classroom • In-class time is spent mainly on coding and problem solving • Online coaching is also used to develop students’ programming skills
Tyler and Abdrakhmanova [81]	• Course content is delivered online • In-class time is spent on collaborative activities that focus on coding and problem solving
Tyler and Yessenbayeva [82]	• Course content is delivered online (video lectures, exercises, and practice quizzes) • Class time was devoted to hands-on work and student evaluation
Uz and Uzun [83]	• Course content is delivered through lectures while labs focus on coding and problem solving • Online content is added as supplementary materials for students to study on their own pace • Students also work collaboratively on a programming project
Wang, Fong [84]	• Course content is delivered through lectures and self-based online resources • Face-to-face tutorials are conducted to allow students to do some programming practices • Computer-assisted instruction system is used to provide an online programming practice platform to students
Yagci [85]	• Course content is delivered through lectures while labs focus on coding and problem solving • Online content is added as supplementary materials for students to study on their own pace • Students were also required to work on groups and have group meetings with their instructors to check their progress
Yagci [86]	• Course content is delivered through lectures and self-based online resources • Online and face-to-face collaborative activates are added for students to discuss and practice coding
Yigit, Koyun [87]	• Course content is delivered online through virtual classes and self-paced resource • Students practice coding individually or by joining small groups • Face-to-face meeting are conducted with the instructor when needed
Yigit, Koyun [88]	• Course content is delivered through lectures and self-based online resources • Students practice coding in the lab as well as online by using an online programming environment
Zampirolli, Goya [89]	• Course content is delivered online through self-paced resource • Each week, students complete a number of exercises that are manually corrected by instructors • Four mandatory face-to-face classes are used to introduce students to the course and evaluate their progress

In fifteen approaches (37%), content delivery was accomplished through both traditional lectures and online resources. Traditional lecturing was the primary method of content delivery in ten approaches (25%), while online learning was the primary method of delivery in fifteen approaches (37%). Practical coding and problem-solving activities took place online in seven approaches (18%), face-to-face in twenty-one approaches (52%) and in both modes in twelve approaches (30%).

### Blended learning components

**Fig 4:**
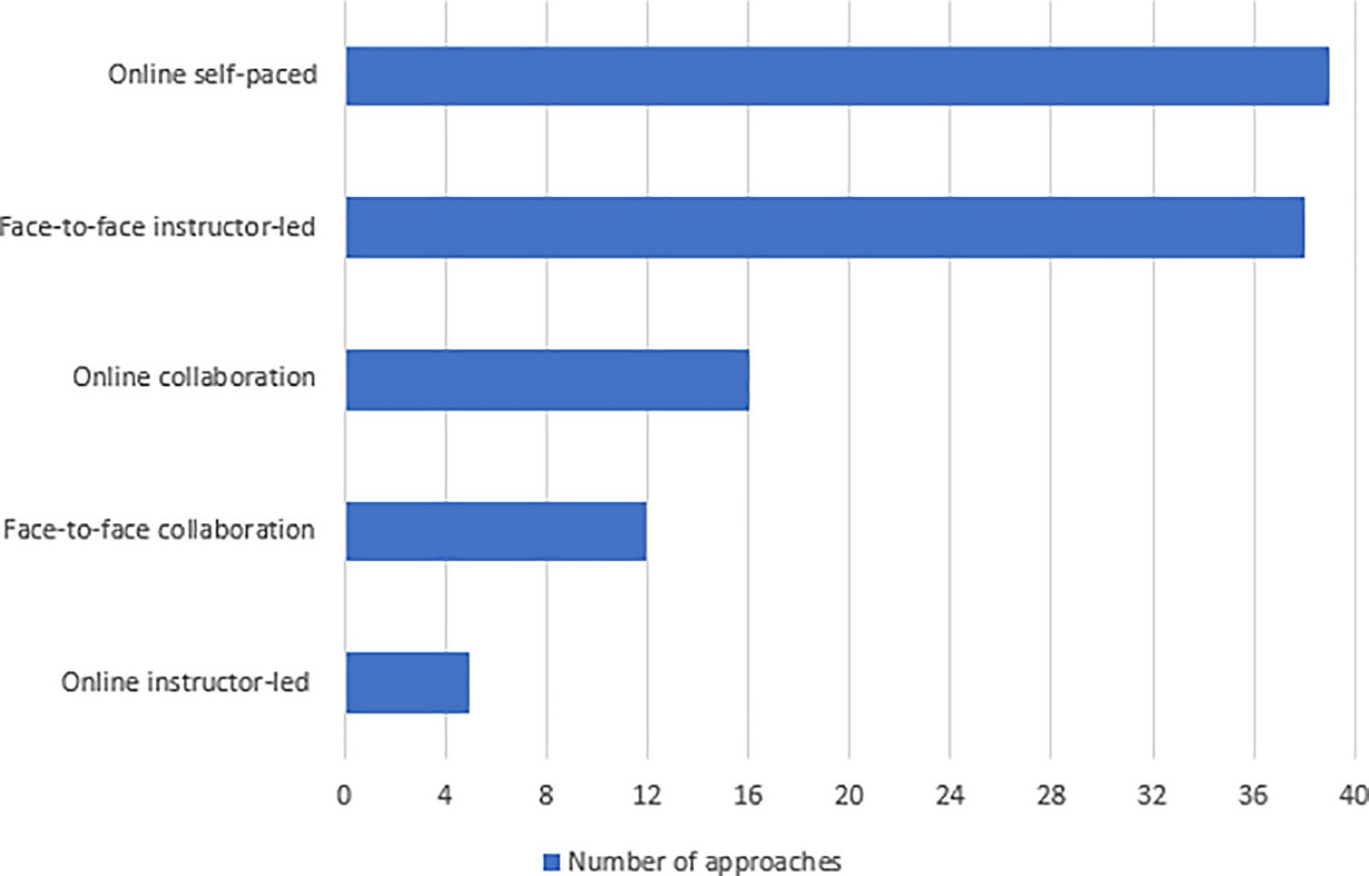
Blended learning components used in the included studies.

As has been discussed in the background section, there are five different blended learning components. Fig 4 shows how many approaches used each of these components. Online self-paced was the most used component (used in 39 out of the 40 approaches), followed by face-to-face instructor-led (used in 38 approaches) then online collaboration (used in 16 approaches). The least used components were face-to-face collaboration (used in 12 approaches) and online instructor-led (used in five approaches only).

### Approaches evaluation

Of the forty approaches identified in this review, thirty-seven of them were evaluated, while the remaining three were not. As can be seen in Table 6, five evaluation criteria were used to evaluate these approaches. The most utilized evaluation criterion was students’ course performance. It was used with thirty approaches. It was measured by examining: pass rate (5 approaches), pre and post-tests (6 approaches), final exam scores (8 approaches), final grades (10 approaches), multiple assessment results (1 approach). Twenty-four studies compared student performance in traditional course to performance in blended courses. The remaining six studies did not, instead it looked at the pass rate as an indicator of students’ performance.

**Table 6:** Evaluation criteria and findings of the reviewed studies.

Criterion	Findings	Approaches
Students’ course performance	Outperformed traditional approach	Cabrera, Villalon [63]*, Timmermann, Kautz [79], Tyler and Abdrakhmanova [81], Dawson, Allen [4]*, Boyle, Bradley [62]*, Clark, Besterfield-Sacre [66], Uz and Uzun [83], Tritrakan, Kidrakarn [80]*, Ortíz-Ortíz, Jiménez-Murillo [72]*, Alhazbi [57]*, Yigit, Koyun [88]*, Djenic, Krneta [75], Wang, Fong [84]*, Álvarez, Martín [59], Deperlioglu and Kose [68]*, Jonsson [71]*
	No difference in compare with traditional approach	Cakiroglu [64], Breimer, Fryling [54]- Half Flip, Breimer, Fryling [54]- Full Flip, Tyler and Yessenbayeva [82], Alonso, Manrique [58], Yigit, Koyun [87], Zampirolli, Goya [89], Sun, Kindy [78]
	High students performance (no comparison)	Albrecht, Gumz [56]*, Hadjerrouit [69], Bati, Gelderblom [6], Hauswirth and Adamoli [55]- Asynchronous, Hauswirth and Adamoli [55]- Synchronous, Yagci [86]*,
Students Satisfaction	Satisfied	Tyler and Abdrakhmanova [81], Dawson, Allen [4], Hadjerrouit [69], Bati, Gelderblom [6], Boyle, Bradley [62], Breimer, Fryling [54]- Half Flip, Breimer, Fryling [54]- Full Flip, Özyurt and Özyurt [74], Impelluso [70], Chen, Li [65], Yagci [86], Uz and Uzun [83], Băutu, Atodiresei [60], Yagci [85], Tritrakan, Kidrakarn [80], Ortíz-Ortíz, Jiménez-Murillo [72], Alhazbi [57], Yigit, Koyun [88], Djenic, Krneta [75], Wang, Fong [84], Bi and Shi [61], Deperlioglu and Kose [68], Jonsson [71]
	Similar satisfaction as compared to traditional course	Alonso, Manrique [58]
	Not satisfied	Clark, Besterfield-Sacre [66]
Student engagement with online resources	Highly engaged	Cabrera, Villalon [63], Tyler and Abdrakhmanova [81], Hauswirth and Adamoli [55]- Asynchronous, Boyle, Bradley [62]
	Reasonably engaged	Albrecht, Gumz [56], Hauswirth and Adamoli [55]- Synchronous, Chen, Li [65]
Support of students’ learning	Supports students learning	Hadjerrouit [69], Dawson, Allen [4], Bati, Gelderblom [6], Davenport [67], Özyurt and Özyurt [74], Chen, Li [65], Uz and Uzun [83], Yagci [85], Ortíz-Ortíz, Jiménez-Murillo [72], Alhazbi [57], Yigit, Koyun [88], Djenic, Krneta [75], Wang, Fong [84], Álvarez, Martín [59]
Students’ learning and programming behaviour	No impact on programming efficiency	Hadjerrouit [69]
	Students spend more time practicing programming	Breimer, Fryling [54]- Half Flip, Breimer, Fryling [54]- Full Flip
	Students spend more time on learning	Alonso, Manrique [58]
	Helped students to develop better programming strategies	Chen, Li [65]

* Indicates significant improvement in students’ course performance

Sixteen approaches were reported to help students outperform their counterparts in the traditional courses. As for eight approaches, researchers found no difference in students’ performance in compare with traditional approaches. For approaches were no comparison was performed (6 approaches), they were all found to help students perform well in the course. Overall, twelve approaches have led to a significant improvement in students’ course performance.

The second most utilized evaluation criterion was students’ satisfaction. It was used to evaluate twenty-five approaches and was measured by questionnaires that asked students to indicate their satisfaction with the blended courses (24 approaches) and interviewing students (1 approach). Students were satisfied with twenty-three approaches, not satisfaction with one approach and indicated similar satisfaction, as compared to traditional course, with one approach.

Support of students’ learning was also one of the evaluation criteria that were utilized in a number of the reviewed studies (14 approaches). It was measured by several qualitative methods: survey (9 approaches), focus group (2 approaches), interview (one approach), informal meeting (one approach) and reflective journals (one approach). All the evaluated approaches (14 approaches) were reported to effectively support students learning.

Another evaluation criterion utilized in some of the reviewed studies is student engagement with online resources. This criterion was evaluated by reviewing students’ online activities, such as logins, videos views, and online forum and wiki posts. Four approaches were reported to significantly enhance students' engagement, while three were found to have reasonable impact on enhancing students’ engagement.

Furthermore, four studies looked at students’ programming and learning behavior to evaluate the applied blended learning approaches. The first study found that the applied approach has no impact on programming efficiency. The second one found that the examined approaches (two approaches) motivated students to spend more time on practicing programming. The third one also found that blended learning approach has led to an increase in the time that students spent on the course. The last study found that the examined approach has helped students to develop their own strategies to solve programming problems.

## Discussion

The publication trend indicates that there is an increasing interest in applying blended learning to introductory programming courses. However, given the importance of the topic and the relatively small number of studies that have been found, more research is needed in this area. Overall, this systematic review of the 38 studies was helpful in answering our three research questions.

### RQ1: What are the different blended learning models that have been applied in introductory programming courses?

As illustrated in the previous section, many different blended learning approaches were applied in introductory programming courses. These approaches can be classified into five models (see Table 7). This classification has been made according to where the content delivery and practical activities take place i.e., face-to-face or online.

**Table 7:** Blended learning models and their corresponding approaches.

Model	Approaches
Flipped	Tyler and Abdrakhmanova [81], Clark, Besterfield-Sacre [66], Breimer, Fryling [54]- Full Flip, Davenport [67], Özyurt and Özyurt [74], Tyler and Yessenbayeva [82], Sun, Kindy [78], Bi and Shi [61], Šarić and Šerić [76], Jonsson [71]
Mixed	Cabrera, Villalon [63], Dawson, Allen [4], Hadjerrouit [69], Boyle, Bradley [62], Breimer, Fryling [54]- Half Flip, Impelluso [70], Yagci [86], Tritrakan, Kidrakarn [80], Alhazbi [57], Yigit, Koyun [88], Djenic, Krneta [75], Wang, Fong [84], Deperlioglu and Kose [68], Othman, Pislaru [73]
Flex	Cakiroglu [64], Hauswirth and Adamoli [55]- Asynchronous, Alonso, Manrique [58], Yigit, Koyun [87], Zampirolli, Goya [89]
Supplemental	Bati, Gelderblom [6], Hauswirth and Adamoli [55]- Synchronous, Chen, Li [65], Djenic and Mitic [77], Uz and Uzun [83], Băutu, Atodiresei [60], Yagci [85], Ortíz-Ortíz, Jiménez-Murillo [72]
Online-practicing	Albrecht, Gumz [56], Timmermann, Kautz [79], Álvarez, Martín [59]

In traditional courses, content delivery and practical activities are both take place face-to-face, while in online courses, they take place online. In blended courses, instructors have the flexibility of conducting them face-to-face, online or in both modes. For face-to-face delivery, they can use any of the two face-to-face components that have been explained in background section i.e. face-to-face instructor-led or face-to-face collaboration. For online delivery, they can use online self-paced, online instructor-led or online collaboration.

Pro-flipped classrooms believe that it is better to do the content delivery online and use class time to work on applications of content, often by using active learning techniques such as problem-based learning or peer instruction [67, 74]. Opponents of this approach argue that students, especially in their first-year of college, do not have the discipline to complete online activities before attending the face-to-face classes. Therefore, this study looked at where content delivery and practical activities occur (face-to-face or online) and use it as a basis for classifying the different blended learning approaches.

### Flipped model

This is probably the most widely known blended learning model. In introductory programming course, it is applied by explaining programming concepts outside the classroom through online resources, then in-class time is spent on active learning that focus on coding and problem solving. Two variations of this model have been identified in this review. In the first one, online work was added to the traditional course without reducing in-class time [81], while in the second one, in-class time was reduced to counterbalance the students' workload [54, 66].

### Mixed model

In this model, content delivery and practical coding activates occur both face-to-face and online. An example of this model is the approach applied by Dawson, Allen [4]. They assigned their students weekly pre-class work consisting of reading materials and solving programming exercises. The face-to-face sessions were then started with mini-lectures to explain difficult programming concepts. After that, students were divided into small groups and were requested to solve programming exercises. Students were also asked to complete online peer review assignments that required them to answer programming problems.

### Flex model

In this model, both content delivery and practical coding activates take place online, but students are required to attend face-to-face sessions from time to time to check their progress or to provide them with feedback. A common way to apply this model is as in [55] who created a complete online course organized into topics. Each topic included a variety of tasks such as: watching a video, reading a book section, participating in an online discussion or solving a programming exercise. Students progressed through these topics at their own pace, while attending weekly face-to-face sessions with their instructors. These sessions were used to check students’ progress and to provide them with feedback.

### Supplemental model

In this model, both content delivery and practical coding activates take place face-to-face. However, online supplemental activities are added to the course to increase students’ engagement with course content [90]. Two variations of this model have been identified in this review. In the first one, online activities were added to the course without connecting them to the in-class activities [6], while in the second one, connections were made between online and in-class activities [55].

### Online-practicing model

In this model, an online programming environment is used as the backbone of students learning. It allows students to practice programming and problem solving; and provides them with immediate feedback about their programming solutions. The delivery of course content is achieved through lectures and/or self-based online resources. In some cases, online resources are integrated within the online programming environment [79].

### RQ2: How effective is blended learning in improving the learning experience of novice programmers?

The vast majority of studies included in this review shows that blended learning has a positive effect on teaching, with students also identifying that blended courses effectively support learning. This is mainly related to the rich variety of face-to-face and online components that could be incorporated into blended courses. As has been discussed in the background section, each of the five components of blended learning has its own advantages. By thoughtful mixing these components, instructors can enhance their students’ learning experience and achieve the desired outcomes.

Integrating online components in introductory programming courses provide students with: higher level of autonomy to organize their learning, better access to learning resources and greater flexibility to plan and manage their study. Albrecht, Gumz [56] and [86] found that online programming environments can be helpful in motivating students to do more programming practice and improving their understanding of programming concepts. In addition, Tyler and Abdrakhmanova [81] reported that interactive videos encourage students to spend more time outside of class on their course work. Furthermore, Hadjerrouit [69] found that online resources were helping students to easily grasp programming concepts, solve programming tasks, reuse program code and revise before examination. In the study conducted by Tritrakan, Kidrakarn [80], students commented that online resources allowed them to have more flexible time to study and understand programming concepts because the time to learn was not limited to face-to-face classes.

In addition, the integration of face-to-face components within introductory programming courses enables students to communicate directly with their instructors and tutors, get direct programming guidance and ask for immediate help when they need it [4, 69]. Face-to-face activities can also help students to engage with each other and develop close associations, which in turn, can help them to communicate better online [63].

Furthermore, a balanced mix of online and face-to-face components enable students to receive knowledge and help from multiple sources and allow instructors to accommodate different learning styles [62]. In the study conducted by Dawson, Allen [4], students commented “we had a lot of chances to learn the material and ask for help. [If] we didn’t understand it during pre-reading we could understand it during class, and then during the peer review, and then during the tutorial, and then during the practice problems”. Similarly, the students who participated in the study of Deperlioglu and Kose [68] noted that after the face-to-face lectures, they understood course topics better with the support of the online interactive contents.

A balanced mix can also help increase the level of active learning strategies such as peer assessments, pair programming, self-assessment quizzes and online discussion [6, 54]. Active learning has proved to help students to develop problem solving and critical thinking skills, and construct a deeper understanding of the programming concepts being taught [66, 74].

### RQ3: Which model is most appropriate for introductory programming courses?

The findings of this systematic review indicate that all the five models have the potential of enhancing the learning experience of novice programmers. However, it seems that the mixed model has the potential of providing even more enhancement to students’ performance. Nine of the fourteen studies that applied the mixed model reported a dramatic improvement in students' performance. This seems to be related to the fact that this model has more flexibility regarding what should go online and what should be taught in the traditional classroom.

Breimer, Fryling [54] reported that not all traditional lecturing can be substituted by video lectures because some students may not learn the different type of materials as effectively from video lectures. They recommended that instructors should use a mix of lectures and self-based online resources for content delivery. Similarly, in an introductory mechanical engineering course offered at Massachusetts Institute of Technology (MIT), the teaching staff started offering the course in a flipped format [91]. They then realized that not all students have the discipline to watch the online videos before attending the face-to-face classes. Therefore, they switched to a mixed format; thus, content not critical to students understanding was explained through online videos while critical content was explained through traditional lectures. This mixed format has resulted in a high student satisfaction and significant improvement in their course performance.

The attitude of first year programming students toward online learning could raise a challenge when applying the flipped, the flex and the online-practicing models. These models rely heavily on online activities as the only mean of content delivery and/or coding practice. Davenport [67], after applying the flipped model, found out that even though students thought the model was beneficial, they indicated later during the semester that short weekly lecture would have helped them to better pick up the required information from the textbook. One of her students commented “I feel topics needed more explanation like in the traditional lecture even if it was just one 30 min lecture after the quiz. It was fine at the beginning but when the topics got harder it became too confusing to self-teach”. Similarly, Zampirolli, Goya [89], while applying the flex model, observed that freshmen students performed poorly in online activities. Therefore, they indicated that it is necessary to monitor students’ online activities to make sure that they are using the learning materials.

Supplemental model could also raise some challenges when utilized in introductory programming courses. A serious issue that could be encountered is that students would approach lectures as substitute for the online materials, and would then not do the online work [82]. This will probably happen when no connection is made between what occurs in class and what happens online [60]. In addition, the application of supplemental model could lead to a phenomenon called “the course-and-a-half syndrome” [92]. This phenomenon happens when instructors add online supplemental components to their traditional courses without eliminating any of the existing activities. As result, course becomes overloaded with tasks and activities and that would negatively affect the students' learning experience [54].

Overall, the findings of this systematic review show that in order to maximize the benefits of any adopted model, several recommendations should be taken into consideration. First, instructors should include a variety of online and face-to-face components in their blend to accommodate different student learning styles and allow students multiple opportunities to learn [4]. Alhazbi [57] found that using a variety of learning activities in his blended course helped him to address diverse learning styles; while some of his students liked online discussion, others were more comfortable with in-class discussion.

The top priority of introductory programming courses is to improve students’ problem-solving skills [78]. To better achieve this goal, a mixture of online and face-to-face activities should be utilized. Interactive videos can be helpful in demonstrating problem-solving steps [87], while face-to-face collaborative activities allow students to learn problem-solving techniques from one another [57]. In-lab coaching can also be utilized to understand students’ needs and provide them with immediate feedback [80]. In addition, students can be given practical exercises to solve individually, later discussing their solutions with peers online [57, 69].

Instructional videos can be useful to help students learn programming concepts. However, these videos should be short (i.e., under 10 minutes) and narrowly focused in content [66]. Many studies have found that student engagement is higher with short videos and that the probability of students watching a video to the end decreases as the video duration increases [56].

Instead of using video captures of in-class lectures, instructors should create videos tailored for online learning. This approach allows greater flexibility in producing high-quality and engaging videos [81]. According to Jonsson [71], well-designed interactive videos can outperform in-person lectures.

Online resources have no value unless students use them. Therefore, instructors should ensure that their students are utilizing these resources, especially those that are the primary means of content delivery [54]. Several techniques can be used for this purpose. For example, instructors can ask their students to complete online [63, 79] or in-class quizzes [67] related to these resources or to submit a summary of what they have learned from them [4]. For videos, instructors can embed questions that pop up to students and have to be answered [56].

Instructors should utilize an online programming environment to encourage students to practice programming at their own pace. This tool should contain different programming tasks and should provide automated assessments of students’ solutions [79, 84]. Students, who participated in the study of Ortíz-Ortíz, Jiménez-Murillo [72], commented that the blended course was a success because of the use of an online programming tool. The tool enabled them to access and practice variety of programming exercises at any time and at their own pace.

A blended course should not be overloaded with activities and tasks. Albrecht, Gumz [56] found that forcing students to do many tasks has no positive impact on their exam performance. Moreover, Breimer, Fryling [54] reported that a high volume of tasks can be overwhelming to students and can badly affect their satisfaction with the course.

The majority of in-class time should be dedicated to active learning activities such as problem solving, pair programming, student discussion, instructor coaching and small group work. According to Hadjerrouit [69], programming is an inherently social activity that students can better develop by interacting with other students and getting feedback from instructors. Clark, Besterfield-Sacre [66] reported that active learning in a blended course allows students to apply the concepts and skills with the instructor present to provide coaching and feedback. At the same time, it improves student engagement and involvement in classrooms.

Blended learning has proved to be an effective approach to improving self-learning skills [78, 85], which are very important in learning programming. Therefore, instructors need to incorporate tasks and activities that help students develop these essential skills. According to Uz and Uzun [83], blended learning is useful in improving students’ self-learning skills because it increases information sharing among them and makes them take responsibilities for their own learning.

Introductory programming students normally find it difficult to adapt to blended learning. To tackle this challenge, early in the semester, students should be made aware of their own learning responsibilities and the technology that they will be using [65, 70]. In addition, they should be provided frequent assessments to gauge progress and make adjustments when necessary [74].

A successful blended course requires continuous review and regular course evaluation. Instructors need to evaluate which of the various blended learning components are helping students to achieve the desired learning outcomes [6]. Components that work well can be improved and the ones that do not must be changed or removed [72]. According to Sun, Kindy [78], continuously improving instructional videos, revising assessment methods, use of up-to-date technology will make the blended course accepted by more students and provide a more effective learning experience.

### Limitations of the systematic review

The limitations of a systematic review are mainly related to three risks: selection bias, publication bias, inaccuracy in data extraction, and misclassification [42]. Although different strategies were adopted to minimize these risks, some potential for bias remains.

Selection bias refers to the tendency of researchers to selectively cite studies that support their own conclusions [93]. This risk was addressed in several ways as has been suggested by Yli-Huumo, Ko [94]. First, a search protocol was carefully designed and applied. second, a pilot search with different keywords was conducted to ensure that the largest possible amount of papers is included in this review. Third, rigorous inclusion and exclusion criteria were defined to ensure that all the included papers are related to the research topic and answer the review questions.

Publication bias refers to the problem that studies with positive results are more likely to be published than the ones with negative results [94]. This issue was addressed by including several well-known scientific databases in the search. This increased the number of papers that were found and to some extent increased the possibility to find papers with negative results. In fact, seven of the thirty-eight studies included in this review reported negative results (i.e., blended learning has no impact on students’ course performance).

Inaccuracy in data extraction and misclassification refer to the likelihood that study’s information is extracted differently by different reviewers [95]. This issue was addressed by asking two independent reviewers to assess all extractions made by the researcher.

## Implications for future research

The findings of this review serve as the basis for recommendations for academics who plan to investigate the role of blended learning in teaching introductory programming courses.

First, all studies included in this review examined the impact of the overall blended experience on students’ course performance. Few studies went a step forward and surveyed students about which components of the blend supported their learning the most. However, none has investigated the actual impact of each of these components on students’ performance. Studying these different components and identifying their strengths and weaknesses can help instructors of introductory programming courses to choose the most suitable components for their blended courses.

Second, the selection of blended learning approaches and the different components of the blend seems to be arbitrary, not evidence-based and possibly not helping instructors to get the maximum benefits of blended learning. Therefore, more researches are needed to investigate and understand how instructors of introductory programming courses are selecting components for their blended courses and whether there are any criteria that should guide their selections.

Third, this systematic review shows that some blended learning approaches rely on online programming tools as the backbone of students learning. It is important to conduct another systematic review to: (i) identify the different tools that are available for instructors to use, and (ii) understand the benefits and challenges associated with each one of these tools.

## Conclusion

The objective of this systematic review was to investigate the different blended learning models that have been applied in introductory programming courses and outcomes associated with them. Five different models were identified: flipped model, mixed model, flex model, supplemental model and online-practicing model. Their classification has been made according to where the content delivery and practical activities occur i.e., face-to-face or online. All these models appear to have the potential of enhancing the learning experience of novice programmers. However, it seems that the flexibility offered by the mixed model can contribute to even better students’ performance. The other four models should be applied with caution. The flipped, the flex and the online-practicing models rely heavily on online components. Therefore, a monitoring strategy should be put in place to ensure that students are doing the online work. For supplemental model, instructors need to make sure that the blended course is not overloaded with activities and that what happens online is well-connected to what occurs in class. Synthesizing the existing research on blending introductory programming courses, this study also provided recommendations for practitioners as well as implications for future studies in the field.

## Supporting information

S1 Checklist PRISMA checklist. (DOC)

S1 Table Evaluation of studies quality. (DOCX)
