# Supplementary material for: Blended learning models for introductory programming courses: A systematic review
Source: PLoS One. 2019 Sep 5;14(9):e0221765. doi: 10.1371/journal.pone.0221765 (PMC6728070; doi:10.1371/journal.pone.0221765)
Supplement: S1 Table — (DOCX) [file pone.0221765.s002.docx]

**S1 Table**. **Evaluation of studies quality.** √ =Criterion met; **×**= Criterion not met

|  | **Outcome measures** | **Background/literature review** | **Sample** | **Study design or methodology** | **Conclusions** | **Overall quality score** |
| --- | --- | --- | --- | --- | --- | --- |
| Cabrera, Villalon, and Chavez (2017) | √ | √ | √ | √ | √ | 5 |
| Timmermann, Kautz, and Skwarek (2016) | √ | √ | √ | √ | √ | 5 |
| Tyler and Abdrakhmanova (2016) | √ | √ | √ | √ | √ | 5 |
| Dawson, Allen, Campbell, and Valair (2018) | √ | √ | √ | √ | √ | 5 |
| Albrecht, Gumz, and Grabowski (2018) | √ | √ | √ | √ | √ | 5 |
| Hauswirth and Adamoli (2017) | √ | √ | √ | √ | √ | 5 |
| Hadjerrouit (2008) | √ | √ | **×** | √ | √ | 4 |
| Bati, Gelderblom, and Van Biljon (2014) | √ | √ | √ | √ | √ | 5 |
| Boyle, Bradley, Chalk, Jones, and Pickard (2003) | √ | √ | **×** | √ | √ | 4 |
| Cakiroglu (2012) | √ | √ | √ | √ | √ | 5 |
| Clark et al. (2016) | √ | √ | √ | √ | √ | 5 |
| Breimer, Fryling, and Yoder (2016) | √ | √ | **×** | √ | √ | 4 |
| Davenport (2018) | √ | **×** | **×** | √ | √ | 3 |
| Özyurt and Özyurt (2017) | √ | √ | **×** | √ | √ | 4 |
| Impelluso (2009) | √ | √ | √ | √ | √ | 5 |
| Chen, Li, and Wang (2012) | **×** | √ | **×** | **×** | √ | 2 |
| Slobodanka Djenic and Mitic (2017) | **×** | √ | **×** | **×** | **×** | 1 |
| Yagci (2018) | √ | √ | √ | √ | √ | 5 |
| Uz and Uzun (2018) | √ | √ | √ | √ | √ | 5 |
| Băutu, Atodiresei, and Băutu (2018) | **×** | √ | **×** | **×** | **×** | 1 |
| Yagci (2017) | √ | √ | **×** | √ | √ | 4 |
| Tritrakan, Kidrakarn, and Asanok (2016) | √ | √ | **×** | √ | √ | 4 |
| Tyler and Yessenbayeva (2018) | **×** | √ | √ | √ | √ | 4 |
| Alonso, Manrique, and Viñes (2009) | √ | √ | √ | √ | √ | 5 |
| Ortíz-Ortíz, Jiménez-Murillo, and Jiménez-Hernández (2018) | √ | √ | √ | √ | √ | 5 |
| Alhazbi (2016) | √ | √ | √ | √ | √ | 5 |
| Yigit, Koyun, Yuksel, Cankaya, and Kose (2015) | **×** | √ | √ | **×** | √ | 3 |
| S Djenic, Krneta, and Mitic (2010) | √ | √ | √ | √ | √ | 5 |
| Wang, Fong, Choy, and Wong (2007) | **×** | √ | **×** | **×** | √ | 2 |
| Álvarez, Martín, Fernández-Castro, and Urretavizcaya (2013) | √ | √ | √ | √ | √ | 5 |
| Yigit, Koyun, Yuksel, and Cankaya (2014) | **×** | √ | **×** | **×** | √ | 2 |
| Zampirolli, Goya, Pimentel, and Kobayashi (2018) | √ | √ | √ | √ | √ | 5 |
| Sun, Kindy, Liron, Grant, and Waterhouse (2012) | √ | √ | √ | √ | √ | 5 |
| Bi and Shi (2019) | **×** | √ | **×** | **×** | √ | 2 |
| Deperlioglu and Kose (2013) | √ | √ | √ | √ | √ | 5 |
| Šarić and Šerić (2018) | **×** | √ | **×** | **×** | √ | 2 |
| Jonsson (2015) | √ | √ | **×** | √ | √ | 4 |
| Othman, Pislaru, and Impes (2013) | **×** | √ | **×** | **×** | **×** | 1 |

Albrecht, E., Gumz, F., & Grabowski, J. (2018). *Experiences in Introducing Blended Learning in an Introductory Programming Course.* Paper presented at the Proceedings of the 3rd European Conference of Software Engineering Education.

Alhazbi, S. (2016). Active blended learning to improve students’ motivation in computer programming courses: A case study. In *Advances in engineering education in the Middle East and North Africa* (pp. 187-204): Springer.

Alonso, F., Manrique, D., & Viñes, J. M. (2009). A moderate constructivist e-learning instructional model evaluated on computer specialists. *Computers & Education, 53*(1), 57-65.

Álvarez, A., Martín, M., Fernández-Castro, I., & Urretavizcaya, M. (2013). Blending traditional teaching methods with learning environments: Experience, cyclical evaluation process and impact with MAgAdI. *Computers & Education, 68*, 129-140.

Bati, T. B., Gelderblom, H., & Van Biljon, J. (2014). A blended learning approach for teaching computer programming: design for large classes in Sub-Saharan Africa. *Computer Science Education, 24*(1), 71-99.

Băutu, E., Atodiresei, A., & Băutu, A. (2018). Design of self-paced blended-learning computer programming courses for maritime students. *Scientific Bulletin" Mircea cel Batran" Naval Academy, 21*(1), 1-7.

Bi, X., & Shi, X. (2019). On the Effects of Computer-Assisted Teaching on Learning Results Based on Blended Learning Method. *International Journal of Emerging Technologies in Learning, 14*(1).

Boyle, T., Bradley, C., Chalk, P., Jones, R., & Pickard, P. (2003). Using blended learning to improve student success rates in learning to program. *Journal of Educational Media, 28*(2-3), 165-178.

Breimer, E., Fryling, M., & Yoder, R. (2016). Full flip, half flip and no flip: Evaluation of flipping an introductory programming course. *Information Systems Education Journal, 14*(5), 4.

Cabrera, I., Villalon, J., & Chavez, J. (2017). Blending Communities and Team-Based Learning in a Programming Course. *IEEE Transactions on Education, 60*(4), 288-295.

Cakiroglu, U. (2012). Comparison of novice programmers’ performances: Blended versus face-to-face. *Turkish Online Journal of Distance Education, 13*(3).

Chen, G.-D., Li, L.-Y., & Wang, C.-Y. (2012). A Community of Practice Approach to Learning Programming. *Turkish Online Journal of Educational Technology-TOJET, 11*(2), 15-26.

Clark, R. M., Besterfield-Sacre, M., Budny, D., Bursic, K. M., Clark, W. W., Norman, B. A., . . . Patzer, J. F. (2016). Flipping Engineering Courses: A School Wide Initiative. *Advances in Engineering Education, 5*(3), n3.

Davenport, C. E. (2018). Evolution in Student Perceptions of a Flipped Classroom in a Computer Programming Course. *Journal of College Science Teaching, 47*(4).

Dawson, J. Q., Allen, M., Campbell, A., & Valair, A. (2018). *Designing an Introductory Programming Course to Improve Non-Majors' Experiences.* Paper presented at the Proceedings of the 49th ACM Technical Symposium on Computer Science Education.

Deperlioglu, O., & Kose, U. (2013). The effectiveness and experiences of blended learning approaches to computer programming education. *Computer Applications in Engineering Education, 21*(2), 328-342.

Djenic, S., Krneta, R., & Mitic, J. (2010). Blended learning of programming in the internet age. *IEEE Transactions on Education, 54*(2), 247-254.

Djenic, S., & Mitic, J. (2017). Teaching Strategies and Methods in Modern Environments for Learning of Programming. *International Association for Development of the Information Society*.

Hadjerrouit, S. (2008). Towards a blended learning model for teaching and learning computer programming: A case study. *Informatics in Education, 7*(2), 181.

Hauswirth, M., & Adamoli, A. (2017). *Metacognitive calibration when learning to program.* Paper presented at the Proceedings of the 17th Koli Calling Conference on Computing Education Research.

Impelluso, T. J. (2009). Leveraging Cognitive Load Theory, Scaffolding, and Distance Technologies to Enhance Computer Programming for Non-Majors. *Advances in Engineering Education, 1*(4), n4.

Jonsson, H. (2015). *Using flipped classroom, peer discussion, and just-in-time teaching to increase learning in a programming course.* Paper presented at the 2015 IEEE Frontiers in Education Conference (FIE).

Ortíz-Ortíz, O., Jiménez-Murillo, J. A., & Jiménez-Hernández, E. M. (2018). *A Web Framework to Improve Computer Programming Learning.* Paper presented at the 2018 IEEE International Autumn Meeting on Power, Electronics and Computing (ROPEC).

Othman, A., Pislaru, C., & Impes, A. (2013). A framework for adopting blended learning in traditional school based learning. *International Journal of Digital Information and Wireless Communications (IJDIWC), 3*(3), 301-318.

Özyurt, Ö., & Özyurt, H. (2017). A qualitative study about enriching programming and algorithm teaching with flipped classroom approach. *Pegem Egitim ve Ogretim Dergisi= Pegem Journal of Education and Instruction, 7*(2), 189.

Šarić, I., & Šerić, L. (2018). *Time Spent Online as an Online Learning Behavior Variable in a Blended Learning Environment with an Ontology-Based Intelligent Tutoring System.* Paper presented at the 2018 26th International Conference on Software, Telecommunications and Computer Networks (SoftCOM).

Sun, L., Kindy, M., Liron, C. C. M., Grant, C., & Waterhouse, S. (2012). Hybrid course design: Leading a new direction in learning programming languages.

Timmermann, D., Kautz, C., & Skwarek, V. (2016). *Evidence-based re-design of an introductory course “programming in C”.* Paper presented at the Frontiers in Education Conference (FIE), 2016 IEEE.

Tritrakan, K., Kidrakarn, P., & Asanok, M. (2016). The Use of Engineering Design Concept for Computer Programming Course: A Model of Blended Learning Environment. *Educational Research and Reviews, 11*(18), 1757-1765.

Tyler, B., & Abdrakhmanova, M. (2016). *Flipping the CS1 and CS2 classrooms in Central Asia.* Paper presented at the Frontiers in Education Conference (FIE), 2016 IEEE.

Tyler, B., & Yessenbayeva, A. (2018). *A Comparison of Flipped Programming Classroom Models–Results by Gender and Major.* Paper presented at the 2018 IEEE Frontiers in Education Conference (FIE).

Uz, R., & Uzun, A. (2018). The Influence of Blended Learning Environment on Self-Regulated and Self-Directed Learning Skills of Learners. *European Journal of Educational Research, 7*(4), 877-886.

Wang, F. L., Fong, J., Choy, M., & Wong, T.-L. (2007). *Blended teaching and learning of computer programming.* Paper presented at the International Conference on Web-Based Learning.

Yagci, M. (2017). A Web-Based Blended Learning Environment for Programming Languages: Students' Opinions. *Journal of Education and Training Studies, 5*(3), 211-218.

Yagci, M. (2018). Impact of the Individual Innovativeness Characteristics on Success and Contentment at the Computer Programming Course: A Web-Based Blended Learning Experience. *Malaysian Online Journal of Educational Technology, 6*(4), 29-39.

Yigit, T., Koyun, A., Yuksel, A. S., & Cankaya, I. A. (2014). Evaluation of blended learning approach in computer engineering education. *Procedia-Social and Behavioral Sciences, 141*, 807-812.

Yigit, T., Koyun, A., Yuksel, A. S., Cankaya, I. A., & Kose, U. (2015). An example application of an artificial intelligence-supported blended learning education program in computer engineering. In *Artificial Intelligence Applications in Distance Education* (pp. 192-210): IGI Global.

Zampirolli, F. A., Goya, D., Pimentel, E. P., & Kobayashi, G. (2018). Evaluation process for an introductory programming course using blended learning in engineering education. *Computer Applications in Engineering Education, 26*(6), 2210-2222.
